# A Case of Gemcitabine-Induced Thrombotic Microangiopathy Treated With Ravulizumab in a Patient With Stage IV Pancreatic Cancer

**DOI:** 10.7759/cureus.13031

**Published:** 2021-01-31

**Authors:** Kira N MacDougall, Benjamin Schwartz, Janine Harewood, Zaheer Bukhari, Elvira Neculiseanu

**Affiliations:** 1 Internal Medicine, Northwell Health, Staten Island, USA; 2 Hematology and Oncology, State University of New York (SUNY) Downstate Health Sciences University, Brooklyn, USA; 3 Pathology, State University of New York (SUNY) Downstate Health Sciences University, Brooklyn, USA

**Keywords:** gemcitabine-induced thrombotic microangiopathy, microangiopathic hemolytic anemia, pancreatic cancer, therapeutic plasma exchange, rituximab, ravulizumab

## Abstract

A 47-year-old male with stage IV pancreatic cancer developed gemcitabine-induced thrombotic microangiopathy (GiTMA) after treatment with gemcitabine and nab-paclitaxel. GiTMA is a rare and life-threatening complication with an incidence ranging from 0.015% to 1.4% and reported mortality rate ranging from 50% to 90%. Clinically, GiTMA manifests as microangiopathic hemolytic anemia, thrombocytopenia, and renal failure. Early identification of GiTMA is essential to initiate early treatment and improve survival. Treatment of GiTMA includes discontinuation of gemcitabine, along with initiation of steroids, therapeutic plasma exchange (TPE), rituximab, and eculizumab. To our knowledge, this is the first case of GiTMA treated with ravulizumab, a long-acting complement inhibitor. Given the increasing number of patients treated with gemcitabine and seriousness of this complication, it is important for physicians to be aware of this disease entity and maintain a high index of suspicion when evaluating patients with microangiopathic hemolytic anemia, thrombocytopenia, and renal failure.

## Introduction

Gemcitabine is a nucleoside analogue of cytarabine that was first approved in 1996 for the treatment of unresectable pancreatic carcinoma. Gemcitabine-induced thrombotic microangiopathy (GiTMA) is a rare, life-threatening disorder of uncontrolled complement activation, characterized by microangiopathic hemolytic anemia, thrombocytopenia, and renal failure. GiTMA represents one subgroup of microangiopathic disorders distinct from idiopathic atypical hemolytic syndrome (aHUS) and acquired idiopathic thrombotic thrombocytopenic purpura (TTP).

GiTMA is a severe disease, with significant morbidity and mortality. Currently, the reported incidence of GiTMA is 0.015%-1.4% [1,2], with mortality rates ranging from 40% to 90% [3]. Given its rarity and potentially devastating consequences, physicians must maintain a high index of suspicion for GiTMA, as early identification is essential to initiate early treatment and improve survival. Here we present a rare case of GiTMA from our institution treated with ravulizumab, and briefly discuss the pathophysiology and treatment options related to this often-lethal complication.

## Case presentation

A 47-year-old male with a past medical history of stage IV pancreatic adenocarcinoma presented to chemotherapy clinic for administration of gemcitabine (Gemzar) and nab-paclitaxel (Abraxane). However, on arrival, he was complaining of fatigue, nausea, epistaxis, and bright red blood per rectum for the past few days. Complete blood count revealed a hemoglobin of 5.4 g/dL and platelet count of 12 × 109/L, and he was promptly referred to the Emergency Department for further evaluation.

With respect to his oncologic history, he initially presented two years prior to an outside hospital with painless jaundice. He was found to have an obstructing mass in the head of the pancreas. He underwent endoscopic retrograde cholangiopancreatography with the placement of a common bile duct stent. Fine needle aspiration of the mass revealed pancreatic adenocarcinoma. He underwent a pancreaticoduodenectomy (Whipple procedure) and pathology revealed a poorly differentiated invasive ductal adenocarcinoma with mucinous features. He was given adjuvant chemotherapy with fluorouracil, oxaliplatin, folinic acid, and irinotecan (FOLFIRINOX) and pegfilgrastim (Neulasta). A follow-up computer tomography (CT) scan showed disease progression with metastasis to the liver. He was switched to second-line therapy with gemcitabine and nab-paclitaxel. He tolerated the first five cycles of this regimen without adverse effects.

**Figure 1:**
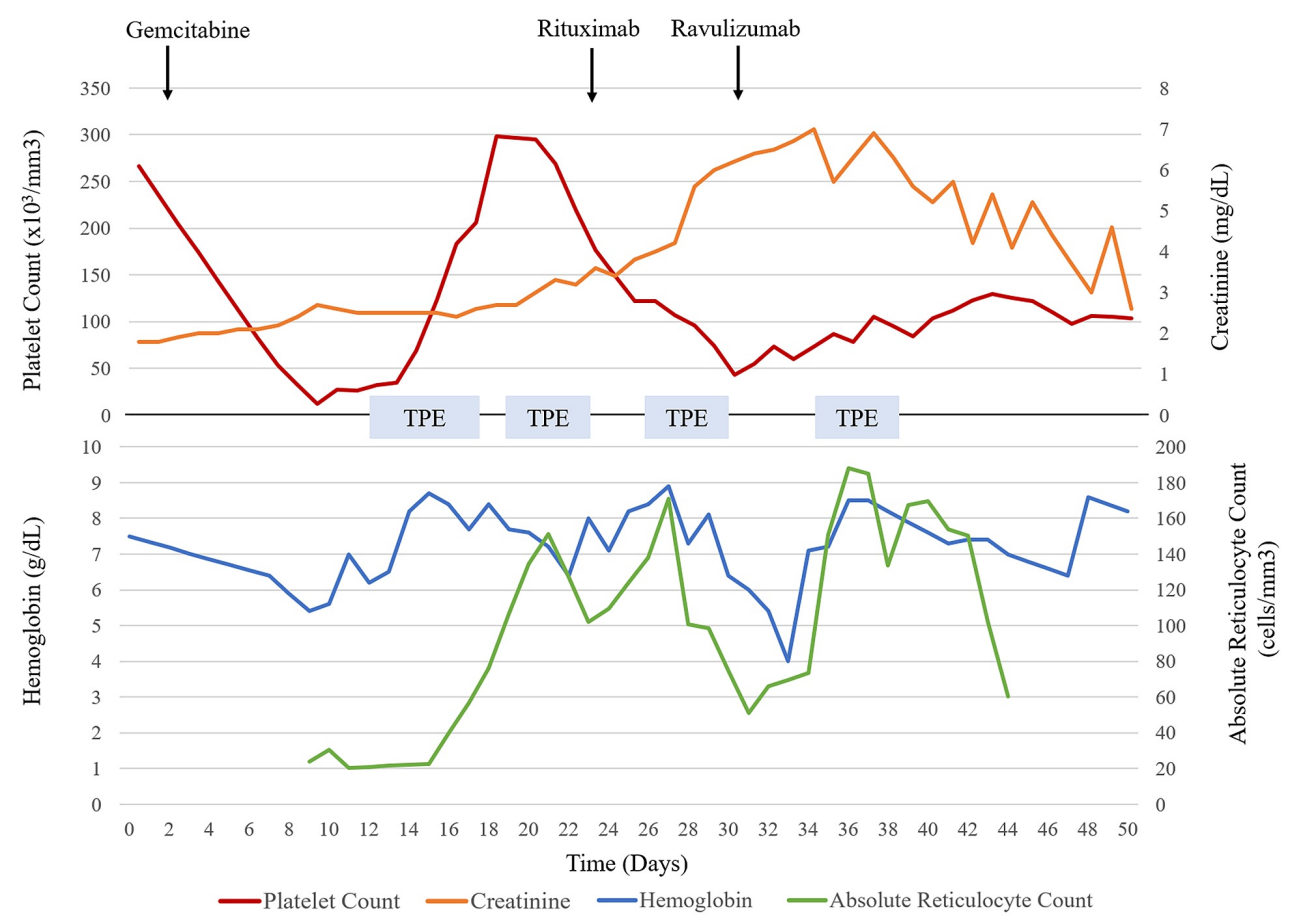
Clinical Course. Trend in hemoglobin, absolute reticulocyte count, platelet count, and serum creatinine over time. Arrows point towards the time of administration of gemcitabine, rituximab, and ravulizumab. TPE = Therapeutic plasma exchange

On admission to the hospital, the patient’s vital signs were within normal limits except for a blood pressure of 191/105 mmHg. The patient did not have a prior history of essential hypertension. His platelets had decreased to 12 × 109/L from a baseline of 266 × 109/L (Figure 1). The patient’s hemoglobin/hematocrit had decreased to 5.4 g/dL (14.8%) from a pre-treatment level of 7.5 g/dL (22.1%). He was also found to have an acute kidney injury with a creatinine of 2.7 mg/dL, from a baseline of 1.8 mg/dL.

**Figure 2:**
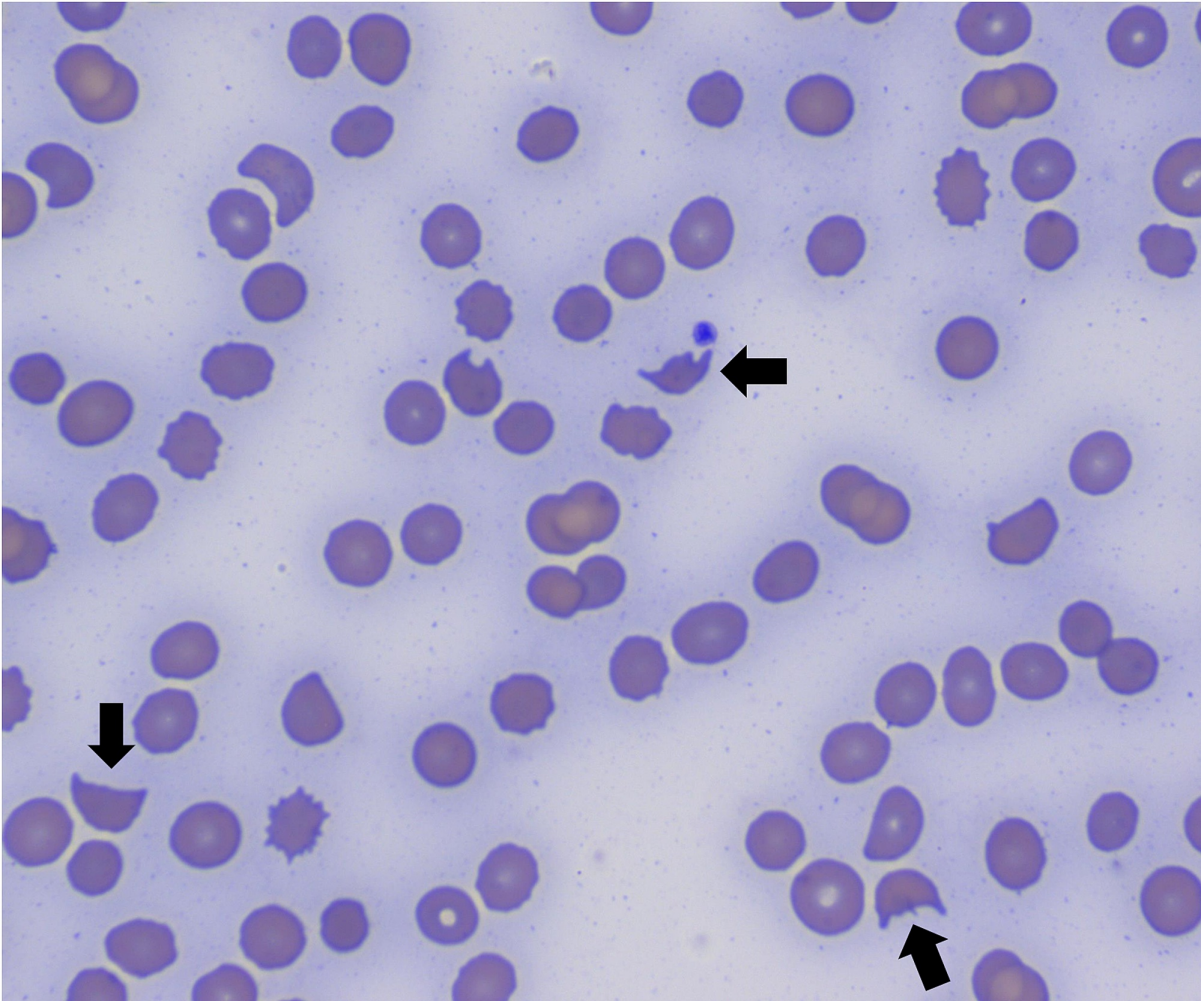
Peripheral blood smear. The arrows indicate schistocytes (fragmented red blood cells with two pointed ends) which were frequently seen in the smear indicating destruction of red blood cells within vascular spaces.

A review of the literature showed that in rare instances, gemcitabine can lead to the life-threatening complication of thrombotic microangiopathic anemia (TMA). A hemolysis panel revealed an elevated lactate dehydrogenase (LDH) of 377 IU/L and haptoglobin of <30 mg/dL. A peripheral blood smear revealed the presence of numerous schistocytes (Figure 2). The coagulation profile was normal with a prothrombin time of 11.8, activated PTT of 25.4, INR of 1.0, and fibrinogen level of 353 mg/dL. The reticulocyte count present and absolute reticulocyte count were 1.41% and 24.0 K/µL, respectively. The reticulocyte index was 0.20. The ADAMTS13 level taken at the time of admission had results and was 56% (normal >68%). Complement C3 was normal at 99 but complement C4 was decreased at 16 (normal 19-52).

The patient was admitted to the medical intensive care unit and was urgently started on therapeutic plasma exchange (TPE) and prednisone (1 mg/kg daily), for possible TTP. After eight days of treatment, his platelets improved to 206 × 109/L. However, over the next week his platelets decreased again to 107 × 109/L. The patient was started on rituximab for refractory GiTMA. After an initial improvement in the patient's hemoglobin, his hemoglobin dropped from 8.9 g/dL to 7.3 g/dL. Despite transfusion with five units of packed red blood cells over the proceeding 48 hours, his hemoglobin dropped further to 6.0 g/dL. His creatinine had also increased from 2.7 mg/dL on admission to 6.4 mg/dL and hemodialysis was initiated. The decision was made to begin therapy with ravulizumab (2,700 mg loading dose), a long-acting complement inhibitor, with initial improvement in the patient’s hematologic parameters and clinical condition.

However, despite treatment with ravulizumab, the patient’s blood counts declined yet again, requiring frequent transfusions of packed red blood cells and platelets. At that point, the patient was no longer a candidate for further chemotherapy, and he was discharged with hospice care.

## Discussion

TMA is a microvascular occlusive disorder characterized by platelet thrombi and glomerular microvascular endothelial injury. The exact pathophysiology of GiTMA is still unclear, but it is suggested that endothelial injury, especially in the kidney, is the central feature [4]. GiTMA is a rare, life-threatening disorder with an incidence ranging from 0.015% to 1.4% [1,2], with mortality rates ranging from 50% to 90% [5].

Early diagnosis of GiTMA is essential to initiate early treatment and improve survival. However, a timely and accurate diagnosis is often complicated by the fact that thrombocytopenia and anemia can occur with myelosuppression, and chemotherapy-induced tubulopathy and microangiopathy caused by disseminated cancer can induce an acute kidney injury [6]. Although an elevated reticulocyte count may be an important clue in most cases of TMA, it may be low in GiTMA secondary to myelosuppression or blood transfusions, as seen in our patient. When the diagnosis of GiTMA is suspected, peripheral blood smear should be screened for the presence of schistocytes and differentiate early-onset GiTMA from myelosuppression. Current molecular tests for mutations or autoantibodies that affect the alternative complement pathway only detect approximately 75% of cases [7].

The variable clinical course of GiTMA further complicates diagnosis. Chemotherapy-induced TMA generally presents four to eight weeks following chemotherapy initiation [8]; however, studies have reported GiTMA up to 10 months after the initiation of gemcitabine [9]. GiTMA can arise after a cumulative dose of 2,450 mg/m^2^ has been administered, but the risk of occurrence increases when the cumulative dose approaches 20,000 mg/m^2^ [4].

New-onset hypertension, as seen in our patient, or exacerbations of existing hypertension, are often seen in GiTMA and may appear before clinical manifestations. One study found that worsening of pre-existing hypertension or new onset of severe hypertension was seen in 90% of patients with GiTMA [10]. Regular visits to the chemotherapy center, where routine vital signs are performed, may provide a unique opportunity to screen patients and allow for early detection of the disease. Heightened awareness of the association between hypertension and GiTMA may lead to earlier diagnosis in some patients.

Uncontrolled activation of the complement system generates the membrane attack complex C5b-9 and anaphylatoxins C3a and C5b [7]. C5b-9 causes swelling of the endothelial cells and is often accompanied by fibrosis. Fibrin-platelet thrombosis occurs where there is a disruption to the endothelial cells. One hypothesis suggests that the kidney is an ideal target for thrombotic microangiopathy because its pH promotes complement activation. This may explain why these patients experience acute kidney injury and in some cases end-stage renal disease requiring dialysis. This theory is supported by autopsy reports demonstrating thrombotic microangiopathy in the kidney.

Discontinuation of gemcitabine is the first step in management. Patients presenting with thrombocytopenia and microangiopathy are often treated with TPE for possible TTP until ADAMTS13 test result. This was the case for our patient. Discontinuation of gemcitabine and TPE alongside steroids are the mainstay of treatment for GiTMA. However, while the efficacy of TPE and steroids for TTP is well established, questions have been raised regarding the efficacy of TPE and steroids for GiTMA [11]. Administration of rituximab has shown benefit in cases where TPE is not rapidly effective [12]. Our patient was also given rituximab with a sub-optimal response.

Eculizumab, an anti-C5 monoclonal antibody, has also shown to be effective in idiopathic (aHUS) [13]. Eculizumab has also been used in the management of GiTMA with significant improvement in both renal function and hematological parameters [5,14-17]. It is often used for patients who are at high risk for complications such as thrombocytopenia, progressive renal failure, or extrarenal complications such as abnormal vascular permeability. Response to therapy further supports the diagnosis. We chose to use ravulizumab for our patient due to its long-acting duration, decreased cost relative to eculizumab, and similar efficacy [18]. To our knowledge, this is the first time ravulizumab has been used to treat GiTMA.

Response to anticomplement therapy depends on the types of complications [19]. Improvement in thrombocytopenia is immediate and a steady increase in platelet count is expected to occur within three days of administration. While our patient did show an improvement within three days, it was not a significant improvement. The reasons for this are likely multifactorial. Firstly, our patient was treated with plasma exchange following administration of ravulizumab due to persistently high reticulocyte count. Secondly, our patient became febrile with positive blood cultures. When compounding factors are simultaneously affecting platelet response, it can pose a unique challenge for physicians caring for these patients. In times of uncertainty, it is recommended to allow time for anticomplement therapy to work before declaring it ineffective. Kidney biopsies have also been used to confirm the presence of TMA [20].

After anticomplement therapy administration, improvement in hypertension is often seen within one to two weeks, and improvement in renal function and hemolysis may not be apparent until weeks to months. Therefore, it is important to allow time for the therapy before declaring it ineffective. Our patient had persistent hemolysis for 10 days following ravulizumab.

For patients with aHUS, it is recommended to check complement total (CH50) before and after each treatment with ravulizumab to confirm complete CH50 suppression. In our case of GiTMA, C3 and C4 were checked prior to administration, which were both within normal limits. CH50 was checked after ravulizumab was given and unexpectedly, was found to be within normal limits. This unexpected finding is not easily explained and further supports the notion that GiTMA is a separate diseases entity and more research is needed to explain the mechanism of this process.

## Conclusions

As the number of patients treated with gemcitabine increases, a heightened awareness of this serious complication is warranted. A high index of suspicion for GiTMA is necessary when patients with gemcitabine present with worsening anemia, thrombocytopenia, and renal failure. Timely discontinuation of gemcitabine, along with administration of steroids, TPE, rituximab, and a complement inhibitor, such as eculizumab or ravulizumab, are warranted. When compounding factors are simultaneously affecting platelet response, it can pose a unique challenge for physicians caring for these patients. In times of uncertainty, it is recommended to allow time for anti-complement therapy to work before declaring it ineffective.

## References

[1] Fung MC, Storniolo AM, Nguyen B, Arning M, Brookfield W, Vigil J (1999). A review of hemolytic uremic syndrome in patients treated with gemcitabine therapy. Cancer.

[2] Müller S, Schütt P, Bojko P (2005). Hemolytic uremic syndrome following prolonged gemcitabine therapy: report of four cases from a single institution. Ann Hematol.

[3] Leal F, Macedo LT, Carvalheira JB (2014). Gemcitabine-related thrombotic microangiopathy: a single-centre retrospective series. J Chemother.

[4] Zupancic M, Shah PC, Shah-Khan F (2007). Gemcitabine-associated thrombotic thrombocytopenic purpura. Lancet Oncol.

[5] Krishnappa V, Gupta M, Shah H (2018). The use of eculizumab in gemcitabine induced thrombotic microangiopathy. BMC Nephrol.

[6] Ryu H, Kang E, Park S, Lee K, Joo KW, Lee H (2015). A case of gemcitabine-induced thrombotic microangiopathy in a urothelial tumor patient with a single kidney. Kidney Res Clin Pract.

[7] Tsai HM (2019). Atypical hemolytic uremic syndrome: beyond hemolysis and uremia. Am J Med.

[8] Van der Heijden M, Ackland SP, Deveridge S (1998). Haemolytic uraemic syndrome associated with bleomycin, epirubicin and cisplatin chemotherapy--a case report and review of the literature. Acta Oncol.

[9] Walter RB, Joerger M, Pestalozzi BC (2002). Gemcitabine-associated hemolytic-uremic syndrome. Am J Kidney Dis.

[10] Glezerman I, Kris MG, Miller V, Seshan S, Flombaum CD (2009). Gemcitabine nephrotoxicity and hemolytic uremic syndrome: report of 29 cases from a single institution. Clin Nephrol.

[11] Gore EM, Jones BS, Marques MB (2009). Is therapeutic plasma exchange indicated for patients with gemcitabine-induced hemolytic uremic syndrome?. J Clin Apher.

[12] Gourley BL, Mesa H, Gupta P (2010). Rapid and complete resolution of chemotherapy-induced thrombotic thrombocytopenic purpura/hemolytic uremic syndrome (TTP/HUS) with rituximab. Cancer Chemother Pharmacol.

[13] Legendre CM, Licht C, Muus P (2013). Terminal complement inhibitor eculizumab in atypical hemolytic-uremic syndrome. N Engl J Med.

[14] Al Ustwani O, Lohr J, Dy G (2014). Eculizumab therapy for gemcitabine induced hemolytic uremic syndrome: case series and concise review. J Gastrointest Oncol.

[15] Rogier T, Gerfaud-Valentin M, Pouteil-Noble C (2016). [Clinical efficacy of eculizumab as treatment of gemcitabine-induced thrombotic microangiopathy: a case report]. (Article in French). Rev Med Interne.

[16] Turner JL, Reardon J, Bekaii-Saab T, Cataland SR, Arango MJ (2016). Gemcitabine-associated thrombotic microangiopathy: response to complement inhibition and reinitiation of gemcitabine. Clin Colorectal Cancer.

[17] Martin K, Roberts V, Chong G, Goodman D, Hill P, Ierino F (2019). Eculizumab therapy in gemcitabine-induced thrombotic microangiopathy in a renal transplant recipient. Oxf Med Case Reports.

[18] (2021). Alexion. ULTOMIRIS® (RAVULIZUMAB-CWVZ). Published.

[19] Tsai HM, Kuo E (2014). Eculizumab therapy leads to rapid resolution of thrombocytopenia in atypical hemolytic uremic syndrome. Adv Hematol.

[20] Katagiri D, Hinoshita F (2018). Gemcitabine-induced thrombotic microangiopathy with nephrotic syndrome. CEN Case Rep.

